# Classification, Prediction, and Concordance of Cognitive and Functional Progression in Patients with Mild Cognitive Impairment in the United States: A Latent Class Analysis

**DOI:** 10.3233/JAD-210305

**Published:** 2021-08-17

**Authors:** Julie Mouchet, Keith A. Betts, Mihaela V. Georgieva, Raluca Ionescu-Ittu, Lesley M. Butler, Xavier Teitsma, Paul Delmar, Thomas Kulalert, JingJing Zhu, Neema Lema, Urvi Desai

**Affiliations:** a F. Hoffmann-La Roche Ltd, Basel, Switzerland; b Analysis Group, Los Angeles, CA, USA; c Analysis Group, Boston, MA, USA; d Groupe d’Analyse, Montréal, QC, Canada; e Analysis Group, London, UK

**Keywords:** Alzheimer’s disease, latent class analysis, mild cognitive impairment, progression

## Abstract

**Background:**

Progression trajectories of patients with mild cognitive impairment (MCI) are currently not well understood.

**Objective:**

To classify patients with incident MCI into different latent classes of progression and identify predictors of progression class.

**Methods:**

Participants with incident MCI were identified from the US National Alzheimer’s Coordinating Center Uniform Data Set (09/2005-02/2019). Clinical Dementia Rating (CDR^®^) Dementia Staging Instrument-Sum of Boxes (CDR-SB), Functional Activities Questionnaire (FAQ), and Mini-Mental State Examination (MMSE) score longitudinal trajectories from MCI diagnosis were fitted using growth mixture models. Predictors of progression class were identified using multivariate multinomial logistic regression models; odds ratios (ORs) and 95% confidence intervals (CIs) were reported.

**Results:**

In total, 21%, 22%, and 57% of participants (*N* = 830) experienced fast, slow, and no progression on CDR-SB, respectively; for FAQ, these figures were 14%, 23%, and 64%, respectively. CDR-SB and FAQ class membership was concordant for most participants (77%). Older age (≥86 versus≤70 years, OR [95% CI] = 5.26 [1.78–15.54]), one copy of *APOE* ɛ4 (1.94 [1.08–3.47]), higher baseline CDR-SB (2.46 [1.56–3.88]), lower baseline MMSE (0.85 [0.75–0.97]), and higher baseline FAQ (1.13 [1.02–1.26]) scores were significant predictors of fast progression versus no progression based on CDR-SB (all *p* < 0.05). Predictors of FAQ class membership were largely similar.

**Conclusion:**

Approximately a third of participants experienced progression based on CDR-SB or FAQ during the  4-year follow-up period. CDR-SB and FAQ class assignment were concordant for the vast majority of participants. Identified predictors may help the selection of patients at higher risk of progression in future trials.

## INTRODUCTION

Alzheimer’s disease (AD) is a debilitating neurodegenerative disorder that greatly impairs the quality of life of patients and close family members [1]. An estimated 5.8 million Americans currently live with AD [2], and this figure is projected to rise to 13.8 million in 2050 mainly due to population aging [3]. Given the high cost related to dementia care [4], AD is expected to impose a growing burden on the United States (US) healthcare system unless a better understanding of AD enables the development of therapies able to halt or slow disease progression. Furthermore, the total number of people with dementia is projected to increase globally in the next decade by 64% (from 50 to 82 million) and to triple by 2050, posing a significant burden on patients and healthcare systems worldwide [5, 6].

There is strong evidence that AD pathological changes begin decades before the emergence of clinical symptoms [7, 8]. Accordingly, current guidelines from the National Institute on Aging/Alzheimer’s Association categorize AD progression into three stages: 1) a preclinical phase characterized by biomarker anomalies, but without cognitive decline [9]; 2) mild cognitive impairment (MCI), which is characterized by short-term and persistent memory problems [10]; and 3) dementia, which is characterized by more severe cognitive and functional impairment [11].

Nonetheless, there is substantial heterogeneity in the rate of progression along this continuum among individual patients with AD [12, 13]. In the 15-year prospective Religious Orders study, 65% of participants exhibited a slow cognitive decline, 27% exhibited a moderate decline, and 8% experienced a rapid decline [13]. The high proportion of patients with slow progression over extended follow-up poses a challenge for the conduct of clinical trials. Therefore, it is critical to document progression trajectories and predictors of progression from MCI to AD in order to help inform the design of future clinical trials by identifying individuals at higher risk of progression for recruitment.

However, the current understanding of progression trajectories among patients with MCI is limited. Previous studies that attempted to identify progression groups included patient populations that were either heterogeneous in terms of disease stage [14] or exclusively focused on patients with dementia [15–17]. Furthermore, there is conflicting evidence on whether cognitive decline precedes functional decline [18–20]; using a variety of instruments and domains to assess patients’ progression early in the AD continuum would be important to shed light on this matter. Therefore, the present study sought to address these limitations and 1) classify patients with incident MCI based on trajectories of cognitive and functional decline, 2) assess the concordance between classes of progression identified using various rating scales, and 3) identify predictors of latent class membership.

## MATERIALS AND METHODS

### Data source

This study used the US National Alzheimer’s Coordinating Center (NACC) Uniform Data Set (UDS) from September 2005 through February 2019. This analysis used data from 26 Alzheimer’s Disease Centers (ADCs) supported by the US National Institute on Aging/National Institutes of Health [21]. Information on participants’ demographics, family history, medical history, cognitive and functional status, behavioral symptoms, and clinical diagnoses of cognitive impairment (including single-clinician or consensus-based determinations) was available [22–24]. Participants enroll at an ADC through referral or as volunteers and may have normal cognition, MCI, or dementia at the time of enrollment. Data were collected prospectively on an approximately annual basis using a standardized clinical evaluation. Measures collected at yearly visits included various measures of cognitive and functional abilities, such as Clinical Dementia Rating (CDR^®^) Dementia Staging Instrument-Global Score (CDR-GS), CDR-Sum of Boxes (CDR-SB), Mini-Mental State Examination (MMSE), and Functional Activities Questionnaire (FAQ).

### Study design and sample selection

The study sample included NACC participants newly diagnosed with MCI. The date of the first MCI diagnosis recorded in the NACC-UDS data was defined as the *index date*. The *follow-up period* was defined as the time from index date until the last observed ADC visit.

Participants were required to meet the following *inclusion criteria*: 1) have a CDR-GS≤0.5 at the time of the MCI diagnosis (patients with CDR-GS score of 0, indicating no cognitive impairment, were allowed as the MCI diagnosis is based on single-clinician or consensus determinations and may be multifactorial), 2) free of MCI≥10 months before the index date (i.e., ≥1 pre-index visit with normal cognition or impaired cognition without MCI), 3) non-missing values for≥1 study outcome on both index visit and≥1 post-index visit, 4) AD etiology on or after the index visit, and 5) no diagnosis of Parkinson’s disease or stroke at any time from the enrollment in the ADC until the end of follow-up.

Eligible participants with≥2 CDR-SB measurements—one at index date and≥1 post-index—were included in the latent class analyses for CDR-SB; similarly, eligible participants with≥2 FAQ measurements at index date and post-index were included in the latent class analyses for FAQ. Concordance between CDR-SB and FAQ class assignments was evaluated among participants included in both the CDR-SB and FAQ analytic cohorts. The analysis of predictors of latent class membership included participants classified in a given CDR-SB or FAQ trajectory who had non-missing values at index date for all candidate predictors.

### Study measures and variables

Study outcomes included measurements of participants’ cognition and function, including CDR-SB and FAQ in the main analyses, and MMSE in sensitivity analyses.

CDR is a comprehensive measure of dementia severity that is commonly used in clinical trials for AD and related dementias. CDR rates performance across six domains associated with cognition and function: memory, orientation, judgement and problem solving, community affairs, home and hobbies, and personal care [25]. The domains are scored independently of one another on a 0 to 3 scale and the category with the highest ranking (i.e., greatest level of impairment) is used to determine the CDR-GS (0 for no dementia, and 0.5, 1, 2, or 3 for questionable, mild, moderate, or severe dementia) [25]. CDR-SB is an additional score, which is the sum of scores from all six domains and thus ranges from 0 to 18, with higher scores reflecting greater impairment. For AD, the CDR-SB recommended cut-off scores are 0.5 to 4.0 for questionable cognitive impairment, 4.5 to 9.0 for mild dementia, 9.5 to 15.5 for moderate dementia, and 16.0 to 18.0 for severe dementia [25, 26]. CDR-SB offers advantages over CDR-GS due to the increased range of values, including increased utility in tracking changes within and between stages of dementia severity [26]. CDR-SB also distinguished MCI from dementia with reasonable accuracy (sensitivity of 71% and specificity of 81%) among patients with a CDR-GS of 0.5 [27].

FAQ was used to measure the participants’ functional status. This instrument quantifies the participants’ ability to perform essential daily activities, such as preparing meals and managing personal finances. The FAQ has a good sensitivity (85%), specificity (81%), and high reliability (> 90%) in distinguishing normal participants from those with dementia [28, 29]. FAQ scores range from 0 to 30, with higher scores reflecting greater functional impairment. While there is some overlap between the FAQ components and the functional domains of the CDR-SB, FAQ is a more comprehensive measure of functional ability.

Cognition measured using the MMSE was assessed as part of a sensitivity analysis [30]. MMSE includes eight domains related to language, repetition, complex commands, orientation to time, orientation to place, registration, attention and calculation, and recall. The scores range from 0 to 30, with lower scores reflecting greater cognitive impairment. This clinical tool was reported to have a sensitivity of≥70%, and a specificity of ≥80% across different settings [31]. The accuracy of baseline MMSE scores for conversion from MCI to AD dementia has ranged from sensitivities of 27% to 89% and specificities from 32% to 90% [32]. MMSE is the most commonly used cognition measure in real-world clinical practice, but since 2015 it was replaced in the NACC UDS by the Montreal Cognitive Assessment (MoCA) test, a similar cognition measure. Where only MoCA scores were available, MoCA scores were mapped into the MMSE scores using a published conversion algorithm [33].

### Statistical analyses

Individual trajectories in CDR-SB and FAQ after the MCI diagnosis were fitted longitudinally using a multi-step growth mixture model (GMM), which classifies participants in latent classes based on the similarity of progression patterns [34, 35]. Given that participants may have occasionally missed a yearly assessment, CDR-SB and FAQ missing data over time were accommodated using full information maximum likelihood under the missing at random assumption [36, 37].

Linear, quadratic, and cubic functional forms were considered for the average CDR-SB and FAQ trajectories in the CDR-SB and FAQ cohorts, respectively. Models with two, three, and four latent classes were then tested. The reported results are based on linear models with three latent classes (fast, slow, and no progression), which were selected based on fit statistics (including Akaike Information Criterion [AIC], Bayesian Information Criterion [BIC], sample-size-adjusted BIC [SABIC], log-likelihood test, and entropy [34, 35, 38]), model parsimony, and sample size for the smallest class (see Supplementary Table 1 for summary statistics). Lower AIC, BIC, SABIC, and log-likelihood values indicate better model fit, while higher values of entropy (> 0.80) indicate better classification quality; additionally, sufficiently large class sizes (smallest class size is at least > 1% of the total count, and/or numerically *n*≥25) are recommended [39].

The overlap in participants’ CDR-SB and FAQ class assignment was evaluated using concordance analyses, which quantified the proportion of participants with identical and different class assignments based on the two measures.

Thirty-one baseline factors identified *a priori* as candidate predictors of progression (listed in Supplementary Tables 2 and 3), based on a targeted literature review and availability in the NACC data, were first included in univariate multinomial logistic regression (MNL) analyses with latent class assignment from the GMM models (i.e., fast versus no progression and slow versus no progression) as dependent variables. All predictors identified as significant at the 5% level from the univariate analyses were subsequently included in the multivariate MNL models from which we report odds ratios (ORs) and 95% confidence intervals (CIs) for all candidate predictors of latent class membership retained from the univariate analyses.

In descriptive analyses, means and standard deviations (SD) were used to describe continuous variables, and frequencies and proportions were used to describe categorical variables.

## RESULTS

### Sample selection and characteristics of study sample

**Fig. 1:**
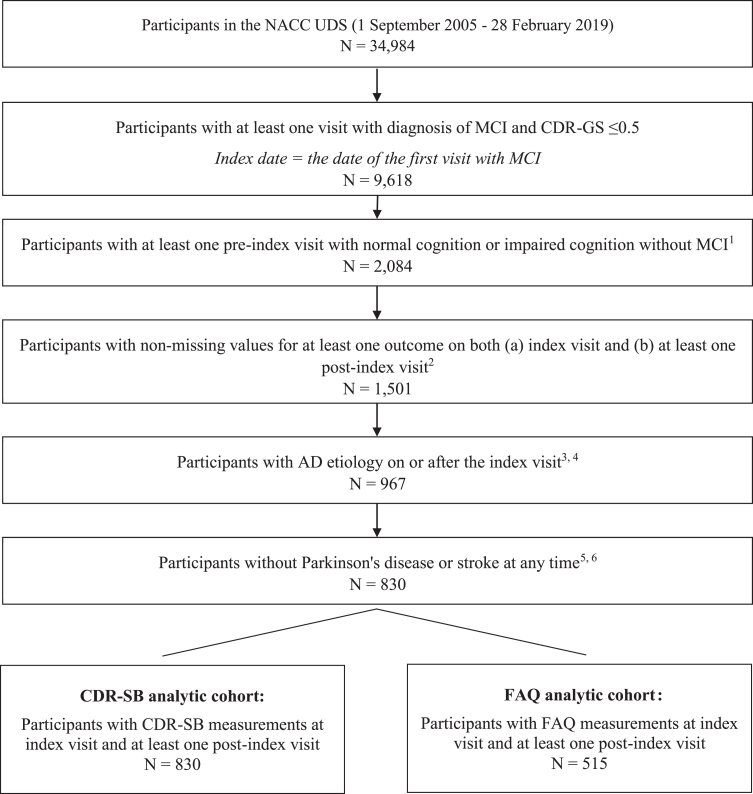
Sample selection flowchart. AD, Alzheimer’s disease; CDR-GS, Clinical Dementia Rating Scale-Global Score; CDR-SB, Clinical Dementia Rating Scale-Sum of Boxes; FAQ, Functional Activities Questionnaire; GDS, Geriatric Depression Scale; MCI, mild cognitive impairment; MMSE, Mini-Mental State Examination; MoCA, Montreal Cognitive Assessment; NACC, National Alzheimer’s Coordinating Center; NPI-Q, Neuropsychiatric Inventory-Questionnaire; UDS, Uniform Data Set. 1) For pre-index visits, only visits that occurred at least 10 months before index visits were considered. 2) Outcomes included: cognition (measured by CDR-SB, CDR-GS, MMSE, MoCA), function (measured by FAQ), behavior (measured by NPI-Q, GDS), and dependency (measured by level of independence). 3) Participants with the following primary etiologies were excluded: Lewy body disease, multiple system atrophy, primary supranuclear palsy, corticobasal degeneration, frontotemporal lobar degeneration, behavioral frontotemporal dementia, primary progressive aphasia, vascular dementia, Down syndrome, Huntington’s disease, prion disease, chronic traumatic encephalopathy, normal-pressure hydrocephalus, epilepsy, CNS neoplasm, bipolar disorder, and schizophrenia/other psychosis. 4) Participants with the following primary or contributing etiologies were excluded: vascular brain injury, essential tremor, traumatic brain injury, and substance abuse (including alcohol). 5) Parkinson’s disease was identified based on disease diagnosis or reported use of Parkinson’s medication, any time during the study period. 6) Stroke was identified based on diagnosis at any time during the study period.

In total, 830 NACC participants newly diagnosed with MCI met the study selection criteria. The mean follow-up time from the index date to the last recorded ADC visit was 3.6 years (SD = 2.5; range: 0–11). Of these, 830 (100%) were included in the CDR-SB analytic cohort, and 515 (62%) were included in the FAQ analytic cohort (Fig. 1).

In the overall study sample, mean age was 79 years, and 58% were females (Table 1). The vast majority (78%) of participants had at least some college education, 78% were non-Hispanic white, and 55% were married. Many patients (61%) had a first degree family relative with cognitive impairment, and 36% had ≥1 copy of the *APOE* ɛ4 allele. Average CDR-SB and FAQ scores at the MCI diagnosis were 0.9 and 1.7, respectively. Memory was the most deteriorated CDR-SB component at MCI diagnosis (mean±SD = 0.5±0.2), and personal care was the least affected component (mean±SD = 0.0±0.1, [Supplementary Table 4]). The three most common comorbidities were hypertension (68%), hyperlipidemia (63%), and depression (34%). The majority of participants had amnestic MCI, either single domain (47%) or multiple domain (38%); only 15% had non-amnestic MCI (single or multiple domain, [Supplementary Table 5]).

**Table 1:** Sample characteristics

	Overall sample *N* = 830	CDR-SB progression classes			FAQ progression classes		
		No progression *N* = 469	Slow progression *N* = 186	Fast progression *N* = 175	No progression *N* = 328	Slow progression *N* = 116	Fast progression *N* = 71
Socio-demographic characteristics
Age (y), mean (SD)	78.5 (8.8)	77.4 (8.5)	79.6 (8.9)	80.2 (9.0)	77.0 (8.1)	79.8 (8.3)	82.2 (9.5)
Male, *n* (%)	347 (41.8)	222 (47.3)	67 (36.0)	58 (33.1)	135 (41.2)	34 (29.3)	18 (25.4)
At least some college education, *n* (%)	646 (77.8)	368 (78.5)	146 (78.5)	132 (75.4)	262 (79.9)	95 (81.9)	52 (73.2)
Non-Hispanic white, *n* (%)	651 (78.4)	363 (77.4)	151 (81.2)	137 (78.3)	263 (80.2)	96 (82.8)	65 (91.5)
Married, *n* (%)	453 (54.6)	262 (55.9)	100 (53.8)	91 (52.0)	171 (52.1)	55 (47.4)	30 (42.3)
Live with spouse/partner, *n* (%)	445 (53.6)	259 (55.2)	97 (52.2)	89 (50.9)	167 (50.9)	55 (47.4)	28 (39.4)
Clinical characteristics
BMI, mean (SD)	26.1 (4.9)	26.3 (4.9)	25.7 (4.8)	25.6 (5.0)	26.3 (4.9)	25.6 (5.1)	24.8 (4.0)
First-degree family member with cognitive impairment, *n* (%)	509 (61.3)	286 (61.0)	116 (62.4)	107 (61.1)	206 (62.8)	81 (69.8)	38 (53.5)
At least one copy of the *APOE* ɛ4 allele, *n* (%)	302 (36.4)	158 (33.7)	66 (35.5)	78 (44.6)	106 (32.4)	48 (41.4)	33 (46.4)
Any FDA-approved AD medication use, *n* (%)^‡^	68 (8.2)	26 (5.5)	17 (9.1)	25 (14.3)	16 (4.9)	10 (8.6)	11 (15.5)
Selected comorbidities
Hypertension, *n* (%)	560 (67.5)	306 (65.2)	127 (68.3)	127 (72.6)	206 (62.8)	73 (62.9)	52 (73.2)
Hyperlipidemia, *n* (%)	525 (63.3)	306 (65.2)	115 (61.8)	104 (59.4)	207 (63.1)	67 (57.8)	43 (60.6)
Depression, *n* (%)	285 (34.3)	146 (31.1)	62 (33.3)	77 (44.0)	104 (31.7)	42 (36.2)	25 (35.2)
Neuropsychiatric disorders, *n* (%)	128 (15.4)	74 (15.8)	28 (15.1)	26 (14.9)	52 (15.9)	14 (12.1)	12 (16.9)
Diabetes, *n* (%)	120 (14.5)	66 (14.1)	24 (12.9)	30 (17.1)	39 (11.9)	14 (12.1)	14 (19.7)
Coronary artery disease, *n* (%)	112 (13.5)	65 (13.9)	21 (11.3)	26 (14.9)	42 (12.8)	11 (9.5)	15 (21.1)
Atrial fibrillation, *n* (%)	89 (10.7)	49 (10.4)	23 (12.4)	17 (9.7)	31 (9.5)	10 (8.6)	6 (8.5)
Traumatic brain injury, *n* (%)	62 (7.5)	37 (7.9)	9 (4.8)	16 (9.1)	27 (8.2)	10 (8.6)	6 (8.5)
Sleep disorders, *n* (%)	52 (6.3)	36 (7.7)	11 (5.9)	5 (2.9)	30 (9.1)	5 (4.3)	3 (4.2)
Congestive heart failure, *n* (%)	34 (4.1)	21 (4.5)	4 (2.2)	9 (5.1)	12 (3.7)	2 (1.7)	3 (4.2)
Cancer, *n* (%)^†^	32 (3.9)	19 (4.1)	8 (4.3)	5 (2.9)	15 (4.6)	4 (3.4)	4 (5.6)
Questionnaire/scale statistics
CDR-SB score at MCI diagnosis, mean (SD)	0.9 (0.8)	0.7 (0.5)	1.1 (0.8)	1.4 (1.0)	0.7 (0.6)	0.9 (0.7)	1.4 (0.9)
FAQ score at MCI diagnosis, mean (SD)	1.7 (3.0)	0.9 (2.0)	2.2 (3.5)	3.4 (4.2)	0.6 (1.5)	1.6 (2.1)	5.3 (4.8)
MMSE score at MCI diagnosis, mean (SD)	27.7 (2.2)	28.0 (2.1)	27.7 (2.2)	26.9 (2.4)	28.2 (1.8)	27.7 (2.1)	26.8 (2.5)
CDR-GS score at MCI diagnosis, mean (SD)	0.4 (0.2)	0.4 (0.2)	0.5 (0.1)	0.5 (0.1)	0.4 (0.2)	0.4 (0.2)	0.5 (0.1)
NPI-Q score at MCI diagnosis, mean (SD)	1.6 (2.7)	1.3 (2.3)	1.8 (2.9)	2.3 (3.3)	1.1 (2.2)	1.5 (2.5)	2.2 (3.5)
GDS score at MCI diagnosis, mean (SD)	2.0 (2.3)	1.9 (2.3)	2.0 (2.3)	2.6 (2.4)	1.7 (2.2)	2.2 (2.3)	2.6 (2.4)
Able to live independently, *n* (%)	756 (91.1)	445 (94.9)	172 (92.5)	139 (79.4)	315 (96.0)	106 (91.4)	59 (83.1)
Follow-up
Years of follow-up, mean (SD)	3.6 (2.5)	3.4 (2.5)	3.9 (2.4)	3.7 (2.3)	3.7 (2.6)	4.7 (2.4)	2.9 (1.6)
Number of NACC visits pre-and post-index date, mean (SD)	7.2 (2.7)	7.1 (2.9)	7.5 (2.7)	7.1 (2.4)	7.1 (2.8)	7.7 (2.6)	7.1 (2.7)

^‡^FDA-approved AD medications include donepezil, galantamine, memantine, and rivastigmine. ^†^A high proportion of participants in the overall sample (77.1%) had unknown cancer status. AD, Alzheimer’s disease; *APOE* ɛ4, ɛ4 allele of the apolipoprotein E gene; BMI, body mass index; CDR-GS, Clinical Dementia Rating Scale- Global Score (scores range from 0–3, with higher values indicating worse cognition; all patients had a score≤0.5 at the MCI diagnosis); CDR-SB, Clinical Dementia Rating Scale-Sum of Boxes (scores range from 0–18, with higher values indicating worse cognition; all patients had a score≤4 at the MCI diagnosis); FAQ, Functional Activities Questionnaire (scores range from 0–30, with higher values indicating worse function); FDA, Food and Drug Administration; GDS, Geriatric Depression Scale (scores range from 0–15, with higher values indicating more advanced depression); MCI, mild cognitive impairment; MMSE, Mini-Mental State Examination (scores range from 0–30, with lower values indicating worse cognition); NPI-Q, Neuropsychiatric Inventory-Questionnaire (scores range from 0–36, with higher values indicating worse neuropsychiatric symptoms); SD, standard deviation.

### Individual progression trajectories observed

**Fig. 2:**
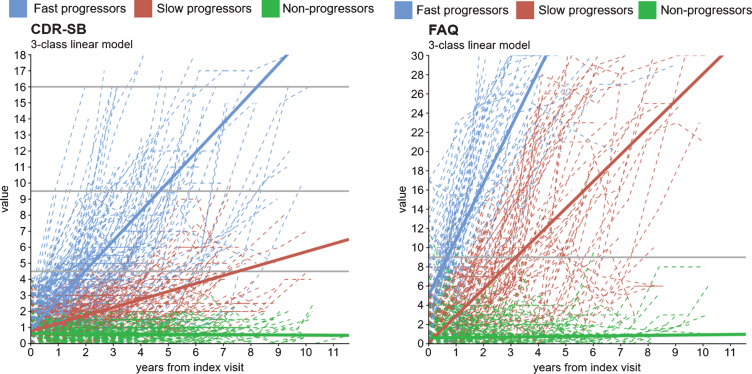
Observed individual and predicted growth trajectories for the 3-class (A) CDR-SB^1^ and (B) FAQ^2^ models^3^. CDR-SB, Clinical Dementia Rating Scale-Sum of Boxes; FAQ, Functional Activities Questionnaire. ^1^ CDR-SB score ranges from 0 to 18 in 0.5 increments. Higher values indicate worse cognition: 0 = Normal; 0.5 –4.0 = Questionable cognitive impairment; 4.5 –9.0 = Mild dementia; 9.5 –15.5 = Moderate dementia; 16.0 –18.0 = Severe dementia. ^2^ FAQ score ranges from 0 to 30. Higher values indicate worse function. A cutoff of 9 (dependent on 3 or more activities) is recommended to indicate impaired function and possible cognitive impairment. ^3^ These models were run using the latent class mixed models (lcmm) package in R. Trajectories are based on lcmm’s predictY function.

Individual observed trajectories of progression are presented in Fig. 2A for CDR-SB and in Fig. 2B for FAQ (dotted lines indicate trajectories of individual patients).

At the time of MCI diagnosis, 101 (12%) participants in the CDR-SB analytic cohort had a CDR-SB score of 0 indicating no cognitive impairment, and 729 (88%) had a CDR-SB score of 0.5–4.0 indicating questionable cognitive impairment. Based on the highest CDR-SB score observed post-MCI diagnosis over an average follow-up of 3.6 years (SD = 2.5), 15% of participants reached a score corresponding to mild dementia (score of 4.5–9.0), 6% reached a score corresponding to moderate dementia (score of 9.5–15.5), and 2% reached a score corresponding to severe dementia (score of 16.0–18.0) [26].

At the time of MCI diagnosis, 495 (96%) participants in the FAQ analytic cohort had an FAQ score < 9 indicating no functional impairment, while 20 participants (4%) had a FAQ score≥9 indicating impaired function and possible cognitive impairment. Over an average follow-up of 3.6 years (SD = 2.5), 158 (19%) participants reached an FAQ score of≥9.

### Latent classes of progression

In the CDR-SB latent class analysis, 21% of participants experienced a fast progression (mean change/year [95% CI] = +1.8 [1.6–2.1] points), 22% experienced a slow progression (mean change/year [95% CI] = +0.5 [0.4–0.6] points), and 57% did not progress (mean change/year [95% CI] = 0.0 [0.0–0.0] points). Figure 2A presents the predicted CDR-SB trajectories for each CDR-SB latent class (solid lines). Similar results were observed in the analysis of MMSE trajectories (Supplementary Figure 1).

In the FAQ latent class analysis, 14% of participants experienced a fast progression (mean change/year [95% CI] = +5.8 [4.9–6.7] points), 23% experienced a slow progression (mean change/year [95% CI] = +2.8 [2.5–3.1] points), and 64% did not progress (mean change/year [95% CI] = 0.0 [0.0–0.0] points). Figure 2B presents the predicted FAQ trajectories for each FAQ latent class.

### Concordance of classes across study measurements

**Fig. 3:**
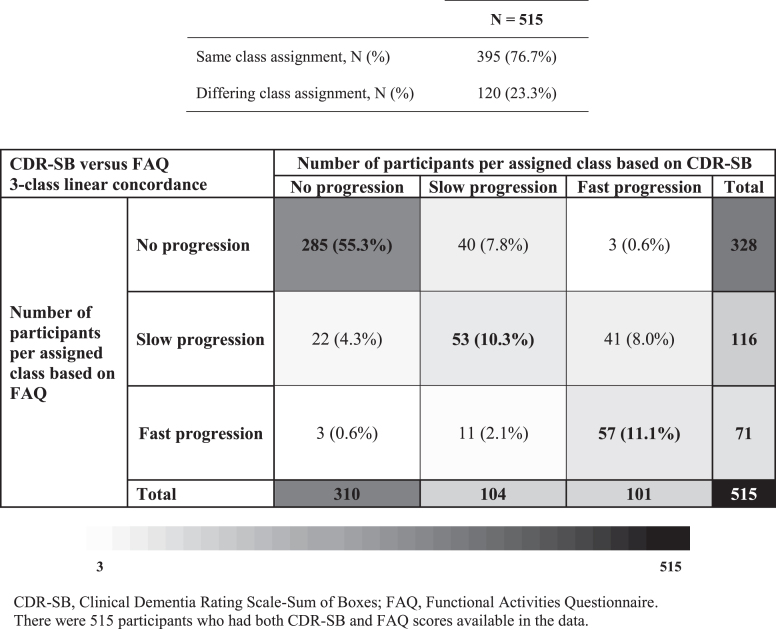
Class assignment concordance between CDR-SB and FAQ 3-class linear models.

Overall, CDR-SB and FAQ class membership was concordant for 77% of participants included in both latent class analyses (*N* = 515; Fig. 3). Large discordances in class membership (i.e., no progression on one scale versus fast progression on the other) were observed in 1% of participants.

Among participants included in both the CDR-SB and MMSE latent class analyses (*N* = 757), class assignment between both measurements was concordant in 56% of participants (Supplementary Figure 2). Large discordances in class membership (i.e., no progression on one scale versus fast progression on the other) were observed in 6% of participants.

### Characteristics of participants stratified by cognitive and functional latent classes

Participants’ age, the proportion of females, and the proportion of participants with≥1 *APOE* ɛ4 allele gradually increased across the three CDR-SB classes of progression (Table 1). The mean baseline CDR-SB score (no progression: 0.7, slow progression: 1.1, fast progression: 1.4), FAQ score (no progression: 0.9, slow progression: 2.2, fast progression: 3.4), and MMSE score (no progression: 28.0, slow progression: 27.7, fast progression: 26.9) gradually worsened across the three latent classes based on CDR-SB. Largely similar trends were observed among the different FAQ (Table 1) and MMSE latent classes (Supplementary Table 6).

### Predictors of class membership

Fifteen candidate predictors were significantly associated with CDR-SB latent class membership in univariate analyses and were included in the multivariate MNL model (Supplementary Table 2). In multivariate analyses, older age (being 76–80 versus ≤70 years and≥86 versus≤70 years), having one copy of *APOE* ɛ4 (versus no copy), having a higher baseline CDR-SB score, and a higher baseline FAQ score were associated with significantly higher odds of being in the fast progression class compared with no progression as measured by CDR-SB (Table 2; all *p* < 0.05). Having a higher baseline MMSE score (i.e., better baseline cognitive status) was associated with lower odds of being in the fast progression class compared with being in the no progression class as measured by CDR-SB. Older age (being 71–75, 76–80, or≥86 years versus≤70 years), having some college (versus graduate school), and having a higher baseline CDR-SB score were associated significantly with greater odds of being in the slow progression class compared with the no progression class based on CDR-SB (all *p* < 0.05). Being 81–85 versus≤70 years was marginally significant (*p* = 0.052).

**Table 2:** Predictors of class membership of progression based on CDR-SB and FAQ, multivariate results

	CDR-SB class membership^1^		FAQ class membership^2^	
	Fast progression versus no progression OR (95% CI)	Slow progression versus no progression OR (95% CI)	Fast progression versus no progression OR (95% CI)	Slow progression versus no progression OR (95% CI)
Age at index date (ref.≤70 y)
71–75 y	2.99 (0.99 –9.06)	**3.09 (1.11 –8.63)**	1.62 (0.27 –9.80)	**3.65 (1.38 –9.63)**
76–80 y	**2.90 (1.02 –8.26)**	**5.21 (2.01 –13.54)**	1.93 (0.36 –10.44)	2.29 (0.89 –5.93)
81–85 y	2.90 (0.99 –8.49)	2.73 (0.99 –7.52)	4.10 (0.78 –21.62)	**4.07 (1.53 –10.81)**
≥86 y	**5.26 (1.78 –15.54)**	**5.57 (2.00 –15.55)**	**8.98 (1.61 –50.22)**	**6.10 (2.17 –17.14)**
Female	1.50 (0.82 –2.76)	1.76 (1.00 –3.11)	1.78 (0.68 –4.69)	**1.78 (1.01 –3.15)**
Marital status (ref. Married/cohabitation)^†^
Widowed	1.52 (0.25 –9.20)	1.09 (0.20 –5.96)	0.36 (0.03 –3.87)	1.66 (0.21 –13.45)
Divorced/separated	0.84 (0.12 –5.78)	0.63 (0.10 –3.84)	0.09 (0.01 –1.34)	0.89 (0.10 –7.73)
Never married	0.50 (0.06 –4.34)	1.79 (0.29 –10.95)	0.06 (0.00 –1.32)	0.78 (0.08 –7.18)
Living situation (ref. Live with spouse/partner)^†^
Live alone	0.96 (0.16 –5.86)	1.07 (0.20 –5.79)	4.06 (0.37 –44.46)	0.71 (0.09 –5.59)
Other (Live with relative/ friend or Live with group)	1.35 (0.20 –9.01)	1.27 (0.21 –7.57)	5.39 (0.46 –63.75)	0.77 (0.09 –6.69)
*APOE* ɛ4 genotype status (ref. No copy)^†^
1 copy	**1.94 (1.08 –3.47)**	1.39 (0.81 –2.41)	**3.28 (1.28 –8.41)**	1.70 (0.97 –2.96)
2 copies	1.55 (0.36 –6.74)	1.56 (0.42 –5.79)	0.98 (0.11 –8.29)	0.85 (0.20 –3.70)
Depression	1.19 (0.61 –2.32)	0.93 (0.49 –1.75)	–	–
Any FDA-approved AD medication use^‡^	2.19 (0.78 –6.14)	2.11 (0.78 –5.71)	1.15 (0.24 –5.54)	1.44 (0.51 –4.05)
CDR-SB score (per point increase)	**2.46 (1.56 –3.88)**	**2.32 (1.49 –3.61)**	0.99 (0.49 –1.99)	1.16 (0.73 –1.83)
CDR-GS score 0.5 (ref. 0)	0.51 (0.21 –1.24)	0.67 (0.30 –1.48)	0.69 (0.15 –3.16)	0.72 (0.33 –1.57)
MMSE score (per point increase)	**0.85 (0.75 –0.97)**	0.94 (0.82 –1.07)	**0.74 (0.61 –0.90)**	0.89 (0.78 –1.02)
FAQ score (per point increase)	**1.13 (1.02 –1.26)**	1.10 (0.98 –1.22)	**1.94 (1.59 –2.36)**	**1.30 (1.12 –1.52)**
NPI-Q score (per point increase)	1.02 (0.91 –1.13)	1.07 (0.97 –1.19)	1.10 (0.93 –1.30)	1.06 (0.95 –1.19)
GDS score (per point increase)	1.04 (0.91 –1.20)	0.93 (0.81 –1.07)	1.04 (0.85 –1.28)	1.04 (0.92 –1.18)
Dependence (ref. Able to live independently)
Requires some assistance with basic or complex activities	1.90 (0.73 –4.94)	0.77 (0.26 –2.28)	0.65 (0.14 –2.94)	0.88 (0.30 –2.64)
Education level (ref. Graduate school)^§^
College	1.63 (0.81 –3.29)	1.16 (0.60 –2.26)	n/a	n/a
Some college	1.33 (0.61 –2.91)	**2.08 (1.04 –4.14)**	n/a	n/a
High school	1.20 (0.52 –2.75)	1.58 (0.76 –3.32)	n/a	n/a
Some high school	0.86 (0.24 –3.15)	0.63 (0.16 –2.40)	n/a	n/a
Ethnicity/race (ref. Non-Hispanic white)^*^
Hispanic/all races	n/a	n/a	0.44 (0.05 –4.11)	1.05 (0.34 –3.24)
Non-Hispanic/African American	n/a	n/a	**0.08 (0.01 –0.79)**	0.41 (0.15 –1.12)
Other^**^	n/a	n/a	0.26 (0.02 –4.36)	0.61 (0.13 –2.84)
BMI^¥^	n/a	n/a	0.98 (0.88 –1.08)	1.02 (0.96 –1.07)

AD, Alzheimer’s disease; *APOE* ɛ4, ɛ4 allele of the apolipoprotein E gene; BMI, body mass index; CDR-GS, Clinical Dementia Rating Scale-Global Score (scores range from 0–3, with higher values indicating worse cognition; all participants had a score≤0.5 at the MCI diagnosis); CDR-SB, Clinical Dementia Rating Scale-Sum of Boxes (scores range from 0–18, with higher values indicating worse cognition; all participants had a score≤4 at the MCI diagnosis); FAQ, Functional Activities Questionnaire (scores ranged from 0–30, with higher values indicating worse function); FDA, Food and Drug Administration; GDS, Geriatric Depression Scale (scores ranged from 0–15, with higher values indicating more advanced depression); MMSE, Mini-Mental State Examination (scores ranged from 0–30, with lower values indicating worse cognition); NPI-Q, Neuropsychiatric Inventory-Questionnaire (scores ranged from 0–36, with higher values indicating worse symptoms); N/A, not applicable; ref., reference group. Bolded estimates are significant at *p* < 0.05. ^†^Unknown categories not shown. ^‡^FDA-approved AD medications include donepezil, galantamine, memantine, and rivastigmine. ^§^Education was identified as a statistically significant predictor in univariate models only for CDR-SB class membership. ^*^Ethnicity/race was identified as a statistically significant predictor in univariate models only for FAQ class membership. ^**^“Other” category for ethnicity/race includes “Non-Hispanic/ Indian or Alaska Native”, “Non-Hispanic/ Asian”, and “Non-Hispanic/Multiracial.” ^¥^BMI was identified as a statistically significant predictor in univariate models only for FAQ class membership. ^1^ 519 observations were included in the CDR-SB complete case analysis. ^2^ 445 observations were included in the FAQ complete case analysis.

Fifteen candidate predictors were significantly associated with FAQ latent class membership in univariate analyses and were included in the multivariate MNL model (Supplementary Table 3). With the exception of education (only a significant predictor for CDR-SB classes) and ethnicity/race and body mass index (only significant for FAQ classes), all other predictors identified in univariate analyses were consistent between the CDR-SB and FAQ classes. In multivariate analyses, predictors associated with significantly higher odds of being in a fast FAQ progression class versus being in the no progression class included older age (being≥86 years versus≤70 years), having one copy of the *APOE* ɛ4 allele (versus no copy), and having a greater baseline FAQ score (all *p* < 0.05). Older age (being 71–75, 81–85, or≥86 years versus≤70 years), female sex, and having a greater baseline FAQ score were associated significantly with greater odds of being in the slow progression class compared with the no progression class based on FAQ (all *p* < 0.05). Having a greater baseline MMSE score was associated with significantly lower odds of being in the fast FAQ progression class (*p* < 0.05) but was not a significant predictor when comparing the slow and no progression classes.

## DISCUSSION

In this retrospective analysis, NACC participants newly diagnosed with MCI were classified in three latent classes of progression (fast, slow, and no progression) based on CDR-SB and FAQ score trajectories. About a third of participants experienced at least some form of progression based on CDR-SB or FAQ during an approximately 4-year follow-up period, whereas the majority of participants were classified in a latent class with minimal or no progression. While CDR-SB assesses both cognition and function, there was a high degree of concordance between CDR-SB and FAQ class membership and a more modest one between CDR-SB and MMSE class membership. The strongest and most consistent predictors of fast progression based on CDR-SB and FAQ identified in multivariate regression analyses were older age; having one copy of the *APOE* ɛ4 allele; and poor baseline CDR-SB, MMSE, and FAQ scores.

Consistent with the present study, most prior studies that assessed trajectories of cognitive and functional scores identified three latent classes of progression [13–15, 40, 41]. A majority of participants included in our study were classified as having either minimal or no progression on CDR-SB or FAQ. Fifteen percent of participants in our sample reached a CDR-SB score corresponding to mild dementia, 6% reached a score corresponding to moderate dementia, and 2% reached a score corresponding to severe dementia over an average follow-up of 3.6 years. This finding is consistent with the updated 2018 MCI treatment guidelines from the American Academy of Neurology, which reported that almost 15% of MCI cases progress to dementia among people 65 years or older followed up for 2 years [42]. Prior studies have also reported that 53%–72% of participants exhibited a modest progression or stable disease trajectory [13–15, 40, 41]. The fact that more than half of patients do not progress over extended follow-up periods makes it more challenging for future trials to detect meaningful benefits conferred by an experimental treatment that slows (i.e., does not reverse) disease progression. Identifying predictors of progression is therefore important to help select subjects with MCI at higher risk of cognitive and functional decline for clinical trial recruitment.

The present study identified several predictors of latent class membership among participants newly diagnosed with MCI. Predictors that were significant for progression on CDR-SB also tended to be significant for progression on FAQ, or at least affected the odds of progression in a similar way. Consistent with several previous studies, older age [40, 43], higher baseline CDR-SB [40, 44], lower baseline MMSE [40, 43], and higher baseline FAQ [18] scores were strong and statistically significant predictors of assignment to the fast progression class. Some studies have previously reported a U-shaped relationship between age at diagnosis and speed of cognitive decline [45, 46]. Rabins et al. found that the youngest and oldest age-of-onset cohorts progressed more rapidly to severe AD than the middle tertile (age 80–86 years) [46]. However, the present study did not find evidence of such an effect. In terms of other predictors of progression, individuals with one copy of the *APOE* ɛ4 allele (versus no copy), but not those with two copies, were at significantly higher risk of progression. This result is consistent with some [47, 48], though not all studies [49–51]. The small number of participants with two copies of the *APOE* ɛ4 allele may explain the absence of impact of gene dosage on the odds of progression.

There was a high degree of concordance between CDR-SB and FAQ class membership, which is consistent with prior studies that found a significant correlation between longitudinal changes in both measurements [52, 53]. This concordance is also likely due to certain overlap between the two measures, with half of the CDR-SB domains being function-related. More than three quarters of participants were classified in concordant CDR-SB and FAQ classes. Discordance in CDR-SB and FAQ class assignment for approximately 25% of participants may in part be explained by mounting evidence that cognitive decline precedes functional decline [19, 20]. Regardless, the strong overlap between CDR-SB and FAQ classes observed in our study suggests that both measures capture similar aspects of progression for the vast majority of participants newly diagnosed with MCI. The concordance between CDR-SB and MMSE trajectory assignment was more modest, with 44% of participants classified in non-concordant latent classes based on both measures. These data suggest that CDR-SB assesses aspects of disease progression that are not fully captured with a less comprehensive instrument such as MMSE. Indeed, a study by Balsis et al. showed that a given MMSE score can be associated with multiple inflections in CDR-SB and Alzheimer’s Disease Assessment Scale-Cognitive Subscale, suggesting the latter instruments are more precise than the former in measuring the severity of cognitive dysfunction [54]. Nevertheless, MMSE and MoCA remain the most commonly used instruments to measure cognition due to their ease of administration and interpretation in real-world clinical practice. A transition towards more comprehensive instruments such as CDR-SB would be beneficial to guide patient management and support future research.

The present study has several strengths. First, in contrast to most previous research, the present study focused exclusively on participants with incident MCI with AD etiology given that the potential clinical benefits of experimental agents are hypothesized to manifest mainly in patients who initiate treatment in the prodromal stage of AD [55]. This group is therefore particularly relevant for the conduct of future trials and for the identification of participants at high risk of subsequent cognitive and functional decline. Second, NACC data are contributed by a network of AD experts practicing in ADCs in the US and allow for a large, well-characterized sample used in the present study. Third, progression was assessed based on both cognitive and functional measures. CDR-SB is a comprehensive measure of dementia severity that incorporates both cognitive and function components; MMSE is a measure of cognitive abilities; and FAQ assesses participants’ routine functional abilities. Fourth, the current study used a data-driven approach to derive progression trajectories; all available data on the outcomes of interest were incorporated in the latent class analyses, thereby limiting potential biases that may ensue from the selection of only participants with complete longitudinal data.

### Limitations

The present study is subject to some limitations. First, study results may not be generalized to the entire US population as many NACC participants represent a clinic-based convenience sample with high education level and may thus not adequately reflect patients encountered in real-world clinical practice. Furthermore, individual ADCs recruit and enroll participants according to their own protocols and do not have required recruitment criteria. As such, the varying inclusion/exclusion criteria may introduce bias into the sample. Second, the National Institute on Aging and the Alzheimer’s Association (NIA-AA) workgroup published their revised criteria for MCI and AD in 2011 [9–11]. However, the 2011 NIA-AA revised criteria were implemented by ADCs only in March 2015. Third, the measures of cognitive and functional assessment used in this study were limited to CDR-SB, MMSE, and FAQ. While MMSE is commonly used in clinical practice, it is not very sensitive to MCI [32]. Further studies are needed to assess progression of MCI to AD using a broader set of neuropsychological tests of cognitive function that extends beyond those that are common in clinical trials. Additionally, all psychometric tests can be subject to floor/ceiling effects. However, CDR-SB was shown to have low levels of floor/ceiling effects, making it a preferred measure for AD clinical trials [56]. Fourth, cerebrospinal fluid and amyloid positron-emission tomography imaging data were not available for most participants, as these are not data elements routinely collected as part of the NACC UDS [57]; in this study, ascertainment of AD as the etiology of MCI was thus based on clinical assessment only. Therefore, results may not be generalized to all patients with MCI, regardless of etiology. Additionally, cerebrospinal fluid and markers of AD pathophysiology (amyloid, tau, and neuronal injury) have been found to predict progression but were not included in the MNL prediction models because biomarker and imaging data are missing for most NACC participants [58, 59]. While the list of predictors was limited to those that are available in the NACC UDS, biomarker confirmation could be an important predictor of progression and further studies are needed to assess its role in disease modifying therapies, particularly among patients with minimally impaired cognition. Fifth, GMM is considered an exploratory data analysis tool, and, given its flexibility, it may identify extraneous latent classes. To mitigate this limitation, the determination of classes was driven not only by fit statistics but also by relevant theory and past research findings as well as model parsimony and interpretability [35, 36]. Lastly, while the exact reason for attrition is not available in the data, participants with worsening cognitive impairment, neuropsychiatric symptoms, and difficulty with functional activities may be more likely to be lost to follow-up [60]. As a result, the long-term decline in all endpoints, particularly in later years post-MCI diagnosis, may be underestimated, given some NACC participants may cease to attend in-person visits at the ADCs when they develop severe AD and move into a long-term care facility.

## CONCLUSION

In this retrospective analysis, participants newly diagnosed with MCI were classified in three latent classes of progression based on CDR-SB and FAQ. Consistent with previous literature, about a third of participants experienced progression on one or both of these scales. The predictors of progression identified in our study contribute to characterizing patients with MCI that may be at higher risk of cognitive and/or functional decline within a relatively short time frame. These patients would be of interest to recruit into a clinical trial for evaluating the efficacy of disease modifying therapies for early AD.

## Supplementary Material

Supplementary Material
