# Supplementary material for: Classification, Prediction, and Concordance of Cognitive and Functional Progression in Patients with Mild Cognitive Impairment in the United States: A Latent Class Analysis
Source: J Alzheimers Dis. 2021 Aug 17;82(4):1667–82. doi: 10.3233/JAD-210305 (PMC8461667; doi:10.3233/JAD-210305)
Supplement: Supplementary Material [file jad-82-jad210305-s001.pdf]

Supplementary Material

Classification, Prediction, and Concordance of Cognitive and Functional Progression in Patients with Mild Cognitive Impairment in the United States: A Latent Class Analysis

Supplementary Table 1. Summary statistics of multi-class linear models[1]

Number of classes	AIC[2]	BIC[2]	SABIC[2]	Log-likelihood[2]	Entropy[3]	Smallest class size, %[4]
CDR-SB latent class models						
2 latent classes	11,861.43	11,908.64	11,876.89	-5,920.71	0.65	32.9%
3 latent classes	11,785.91	11,852.01	11,807.55	-5,878.95	0.53	21.1%
4 latent classes	11,730.48	11,815.46	11,758.3	-5,847.24	0.62	2.3%
FAQ latent class models						
2 latent classes	10,604.97	10,647.41	10,615.67	-5,292.49	0.75	35.2%
3 latent classes	10,559.63	10,619.05	10,574.61	-5,265.82	0.71	13.8%
4 latent classes	10,554.05	10,630.45	10,573.31	-5,259.03	0.72	6.6%
MMSE latent class models						
2 latent classes	13,409.03	13,455.32	13,423.57	-6,694.51	0.61	28.5%
3 latent classes	13,299.95	13,364.76	13,320.3	-6,635.97	0.59	13.1%
4 latent classes	13,279.74	13,363.07	13,305.91	-6,621.87	0.53	9.5%

AIC, Akaike Information Criterion; BIC, Bayesian Information Criterion; CDR-SB, Clinical Dementia Rating Scale-Sum of Boxes; FAQ, Functional Activities Questionnaire; MMSE, Mini-Mental State Examination; SABIC, sample-size-adjusted BIC.

[1] Other functional forms (i.e., quadratic and cubic models) were not considered due to suboptimal separation of progression classes.

[2] Lower AIC, BIC, SABIC, and log-likelihood values indicate better model fit.

[3] Higher values of entropy (>0.80) indicate better classification quality.

[4] In a given class there should be no less than 1% of the total count.

Supplementary Table 2. Predictors of CDR-SB class membership of progression, univariate results

	Slow progression versus No progression		Fast progression versus No progression	
	OR (95% CI)	p	OR (95% CI)	p
**Candidate predictor**				
Age at index date (ref: ≤70 y)				
71-75	**1.95 (1.02 - 3.73)**	**0.044**	1.17 (0.60 - 2.26)	0.646
76-80 y	**2.31 (1.27 - 4.20)**	**0.006**	1.45 (0.80 - 2.62)	0.219
81-85 y	**2.31 (1.27 - 4.20)**	**0.006**	1.74 (0.98 - 3.10)	0.060
≥86 y	**3.18 (1.72 - 5.85)**	**0.000**	**3.08 (1.74 - 5.45)**	**0.000**
Female (ref: male)	**1.60 (1.12 - 2.27)**	**0.009**	**1.81 (1.26 - 2.61)**	**0.001**
Education level[1] (ref: Graduate school)				
College	1.28 (0.81 - 2.02)	0.289	1.46 (0.92 - 2.31)	0.106
Some college	**1.74 (1.08 - 2.79)**	**0.023**	1.39 (0.83 - 2.33)	0.204
High school	1.34 (0.80 - 2.26)	0.271	1.51 (0.90 - 2.55)	0.122
Some high school	0.94 (0.44 - 2.02)	0.872	1.13 (0.53 - 2.37)	0.756
Ethnicity/ race (ref: Non-Hispanic/White)				
Hispanic/all races	0.95 (0.48 - 1.85)	0.873	1.36 (0.74 - 2.53)	0.323
Non-Hispanic/African	0.71 (0.40 - 1.28)	0.259	0.83 (0.47 - 1.49)	0.540
Other[2]	0.76 (0.30 - 1.94)	0.564	0.56 (0.19 - 1.67)	0.297
Marital status[1] (ref: Married/Cohabitation)				
Widowed	1.38 (0.92 - 2.07)	0.116	**1.79 (1.21 - 2.66)**	**0.004**
Divorced/Separated	**0.47 (0.25 - 0.91)**	**0.026**	0.66 (0.36 - 1.21)	0.176
Never married	1.34 (0.73 - 2.47)	0.348	0.41 (0.16 - 1.09)	0.073
Living situation[1] (ref: Live with spouse/partner				
Live alone	1.10 (0.77 - 1.58)	0.598	1.04 (0.71 - 1.51)	0.854
Other[3]	1.38 (0.71 - 2.69)	0.342	**2.01 (1.08 - 3.73)**	**0.027**
First-degree family member with cognitive impairment[1]	1.17 (0.80 - 1.71)	0.415	1.00 (0.69 - 1.46)	0.982
APOE ε4 genotype status[1] (ref: no copy)				
1 copy	1.12 (0.77 - 1.64)	0.553	**1.61 (1.11 - 2.35)**	**0.013**
2 copies	1.18 (0.47 - 2.95)	0.722	1.64 (0.68 - 3.95)	0.275
BMI (per point increase)	0.97 (0.94 - 1.01)	0.184	0.97 (0.93 - 1.01)	0.145
History of smoking[1] (ref: Always non-smoker)				
Past smoker	0.82 (0.58 - 1.16)	0.271	0.87 (0.61 - 1.23)	0.420
Current smoker	2.08 (0.78 - 5.55)	0.143	0.55 (0.12 - 2.58)	0.446
Current alcohol abuse	1.27 (0.43 - 3.76)	0.669	2.49 (0.99 - 6.23)	0.052
Atrial fibrillation	1.21 (0.71 - 2.05)	0.480	0.92 (0.52 - 1.65)	0.785
Cancer[1]	1.73 (0.68 - 4.37)	0.246	1.46 (0.49 - 4.36)	0.497
Congestive heart failure	0.47 (0.16 - 1.38)	0.170	1.16 (0.52 - 2.58)	0.722
Coronary artery disease	0.79 (0.47 - 1.34)	0.381	1.08 (0.66 - 1.77)	0.746

Diabetes	0.90 (0.55 - 1.49)	0.695	1.26 (0.79 - 2.02)	0.331
Depression	1.11 (0.77 - 1.59)	0.585	**1.74 (1.22 - 2.48)**	**0.002**
Hyperlipidemia	0.86 (0.61 - 1.23)	0.411	0.78 (0.55 - 1.11)	0.173
Hypertension	1.15 (0.80 - 1.65)	0.460	1.41 (0.96 - 2.07)	0.079
Neuropsychiatric disorders	0.95 (0.59 - 1.52)	0.818	0.93 (0.57 - 1.51)	0.774
Sleep disorders	1.25 (0.55 - 2.84)	0.594	0.74 (0.25 - 2.14)	0.573
Traumatic brain injury[1]	0.52 (0.24 - 1.10)	0.087	0.97 (0.52 - 1.81)	0.935
Any medication use at MCI diagnosis (ref: No medication use) §: Yes	1.70 (0.56 - 5.11)	0.346	1.61 (0.53 - 4.86)	0.397
Any FDA-approved AD medication use (ref: No medication use)	1.71 (0.91 - 3.24)	0.097	**2.84 (1.59 - 5.07)**	**0.000**
CDR-SB score[4] (per point increase)	**2.45 (1.88 - 3.18)**	**0.000**	**3.72 (2.85 - 4.84)**	**0.000**
CDR-GS score[5] 0.5 (ref. 0)	**2.23 (1.29 - 3.87)**	**0.004**	**3.05 (1.62 - 5.73)**	**0.001**
MMSE score[6] (per point increase)	0.94 (0.86 - 1.02)	0.117	**0.83 (0.77 - 0.89)**	**0.000**
FAQ score[7] (per point increase)	**1.22 (1.12 - 1.32)**	**0.000**	**1.31 (1.21 - 1.42)**	**0.000**
NPI-Q score[8] (per point increase)	1.07 (1.00 - 1.14)	0.052	**1.13 (1.06 - 1.21)**	**0.000**
GDS score[9] (per point increase)	1.04 (0.96 - 1.12)	0.348	**1.13 (1.05 - 1.21)**	**0.001**
Dependence - Requires some assistance with basic or complex activities (ref: Able to live independently)	1.51 (0.76 - 2.99)	0.237	**4.80 (2.77 - 8.33)**	**0.000**

AD, Alzheimer's disease; *APOE* ε4, apolipoprotein E for the ε4 allele; BMI, body mass index; CDR-GS, Clinical Dementia Rating Scale-Global Score; CDR-SB, Clinical Dementia Rating Scale-Sum of Boxes; CI, confidence interval; FAQ, Functional Activities Questionnaire; FDA, Food and Drug Administration; GDS, Geriatric Depression Scale; MMSE, Mini-Mental State Examination; MoCA, Montreal Cognitive Assessment; NPI-Q, Neuropsychiatric Inventory-Questionnaire; OR, odds ratio.

[1] The results for the "Unknown" category are not reported due to their lack of interpretability.

[2] "Other" category for ethnicity/race includes "Non-Hispanic/ Indian or Alaska Native", "Non-Hispanic/ Asian", and "Non-Hispanic/ Multiracial."

[3] "Other" category for living situation includes "Live with relative/friend" and "Live with group."

[4] CDR-SB is scored on a scale of 0-18.

[5] CDR-GS is scored on a scale of 0-3. By design, only values 0 and 0.5 are observed at index date.

[6] MMSE is scored on a scale of 0-30. MoCA scores have been mapped into the MMSE scores using a published conversion algorithm (Trzepacz, 2015). MMSE is the only studied measure in which lower scores indicate worse outcome.

[7] FAQ is scored on a scale of 0-30.

[8] NPI-Q is scored on a scale of 0-36.

[9] GDS is scored on a scale of 0-15.

Supplementary Table 3. Predictors of FAQ class membership of progression, univariate results

	Slow progression versus No progression		Fast progression versus No progression	
	**OR (95% CI)**	**p**	**OR (95% CI)**	**p**
**Candidate predictor**				
Age at index date (ref: ≤70 y)				
71-75	**2.73 (1.23 - 6.06)**	**0.014**	1.30 (0.46 - 3.69)	0.617
76-80 y	1.93 (0.88 - 4.24)	0.100	1.04 (0.38 - 2.84)	0.940
81-85 y	**2.65 (1.23 - 5.74)**	**0.013**	1.96 (0.78 - 4.90)	0.152
≥86 y	**4.11 (1.89 - 8.94)**	**0.000**	**5.66 (2.40 - 13.32)**	**0.000**
Female (ref: male)	**1.69 (1.07 - 2.66)**	**0.025**	**2.06 (1.16 - 3.67)**	**0.014**
Education level[1] (ref: Graduate school)				
College	0.97 (0.55 - 1.70)	0.908	0.88 (0.42 - 1.84)	0.734
Some college	1.71 (0.96 - 3.04)	0.068	1.82 (0.90 - 3.71)	0.097
High school	1.04 (0.53 - 2.03)	0.904	1.89 (0.92 - 3.91)	0.084
Some high school	0.92 (0.32 - 2.65)	0.876	0.67 (0.14 - 3.10)	0.607
Ethnicity/ race (ref: Non-Hispanic/ White)				
Hispanic/all races	1.10 (0.41 - 2.91)	0.854	0.81 (0.23 - 2.88)	0.744
Non-Hispanic/African American	0.70 (0.34 - 1.46)	0.345	**0.21 (0.05 - 0.88)**	**0.033**
Other[2]	1.00 (0.31 - 3.20)	0.995	0.37 (0.05 - 2.90)	0.342
Marital status[1] (ref: Married/ Cohabitation)				
Widowed	1.51 (0.92 - 2.46)	0.101	**2.71 (1.56 - 4.72)**	**0.000**
Divorced/Separated	0.75 (0.37 - 1.50)	0.414	0.47 (0.16 - 1.41)	0.179
Never married	0.76 (0.32 - 1.84)	0.547	0.21 (0.03 - 1.58)	0.129
Living situation[1] (ref: Live with spouse/partner)				
Live alone	1.09 (0.70 - 1.70)	0.697	1.33 (0.76 - 2.32)	0.317
Other[3]	1.59 (0.72 - 3.51)	0.250	**2.84 (1.21 - 6.66)**	**0.016**
First-degree family member with cognitive impairment[1]	1.42 (0.88 - 2.31)	0.153	0.65 (0.38 - 1.10)	0.108
APOE ε4 genotype status[1] (ref: no copy)				
1 copy	1.59 (1.00 - 2.53)	0.052	**1.83 (1.05 - 3.17)**	**0.033**
2 copies	1.32 (0.45 - 3.86)	0.612	1.80 (0.55 - 5.85)	0.328
BMI (per point increase)	0.97 (0.93 - 1.02)	0.227	**0.93 (0.87 - 1.00)**	**0.041**
History of smoking[1] (ref: Always non-smoker)				
Past smoker	0.86 (0.56 - 1.33)	0.508	0.73 (0.43 - 1.24)	0.245
Current smoker	0.52 (0.11 - 2.43)	0.406	1.21 (0.32 - 4.61)	0.775
Current alcohol abuse	0.94 (0.25 - 3.54)	0.928	0.51 (0.06 - 4.06)	0.522
Atrial fibrillation	0.90 (0.43 - 1.91)	0.791	0.88 (0.35 - 2.21)	0.792
Cancer[1]	1.58 (0.45 - 5.51)	0.474	2.28 (0.62 - 8.38)	0.214

Congestive heart failure	0.46 (0.10 - 2.10)	0.317	1.16 (0.32 - 4.23)	0.820
Coronary artery disease	0.71 (0.35 - 1.44)	0.345	1.82 (0.95 - 3.51)	0.072
Diabetes	1.02 (0.53 - 1.95)	0.959	1.82 (0.93 - 3.57)	0.081
Depression	1.22 (0.78 - 1.91)	0.376	1.17 (0.68 - 2.01)	0.567
Hyperlipidemia	0.80 (0.52 - 1.23)	0.309	0.90 (0.53 - 1.52)	0.688
Hypertension	1.01 (0.65 - 1.56)	0.981	1.62 (0.92 - 2.87)	0.097
Neuropsychiatric disorders	0.73 (0.39 - 1.37)	0.326	1.08 (0.54 - 2.15)	0.827
Sleep disorders	1.02 (0.32 - 3.24)	0.978	0.61 (0.16 - 2.38)	0.477
Traumatic brain injury[1]	0.87 (0.40 - 1.87)	0.721	0.89 (0.35 - 2.28)	0.813
Any medication use at MCI diagnosis (ref: No medication use)	2.16 (0.48 - 9.82)	0.317	2.66 (0.34 - 20.78)	0.351
Any FDA-approved AD medication use (ref: No medication use)	1.84 (0.81 - 4.18)	0.145	**3.58 (1.58 - 8.08)**	**0.002**
CDR-SB score[4] (per point increase)	**1.61 (1.18 - 2.21)**	**0.003**	**2.96 (2.12 - 4.13)**	**0.000**
CDR-GS score[5] 0.5 (ref. 0)	1.60 (0.89 - 2.90)	0.117	**5.82 (1.78 - 19.06)**	**0.004**
MMSE score[6] (per point increase)	**0.87 (0.78 - 0.97)**	**0.010**	**0.74 (0.66 - 0.83)**	**0.000**
FAQ score[7] (per point increase)	**1.35 (1.20 - 1.52)**	**0.000**	**1.81 (1.59 - 2.06)**	**0.000**
NPI-Q score[8] (per point increase)	1.07 (0.98 - 1.17)	0.115	**1.15 (1.05 - 1.26)**	**0.002**
GDS score[9] (per point increase)	1.09 (0.99 - 1.19)	0.072	**1.16 (1.04 - 1.28)**	**0.006**
Dependence - Requires some assistance with basic or complex activities (ref: Able to live independently)	2.29 (0.97 - 5.37)	0.058	**4.93 (2.14 - 11.33)**	**0.000**

AD, Alzheimer's disease; APOE ε4, apolipoprotein E for the ε4 allele; BMI, body mass index; CDR-GS, Clinical Dementia Rating Scale-Global Score; CDR-SB, Clinical Dementia Rating Scale-Sum of Boxes; CI, confidence interval; FAQ, Functional Activities Questionnaire; FDA, Food and Drug Administration; GDS, Geriatric Depression Scale; MMSE, Mini-Mental State Examination; MoCA, Montreal Cognitive Assessment; NPI-Q, Neuropsychiatric Inventory-Questionnaire; OR: odds ratio.

[1] The results for the "Unknown" category are not reported due to their lack of interpretability.

[2] "Other" category for ethnicity/race includes "Non-Hispanic/ Indian or Alaska Native", "Non-Hispanic/ Asian", and "Non-Hispanic/ Multiracial."

[3] "Other" category for living situation includes "Live with relative/friend" and "Live with group."

[4] CDR-SB is scored on a scale of 0-18.

[5] CDR-GS is scored on a scale of 0-3. By design, only values 0 and 0.5 are observed at index date.

[6] MMSE is scored on a scale of 0-30. MoCA scores have been mapped into the MMSE scores using a published conversion algorithm (Trzepacz, 2015). MMSE is the only studied measure in which lower scores indicate worse outcome.

[7] FAQ is scored on a scale of 0-30.

[8] NPI-Q is scored on a scale of 0-36.

[9] GDS is scored on a scale of 0-15.

Supplementary Table 4. CDR-SB components at index among the overall sample and progression classes

	Overall sample	CDR-SB progression classes			FAQ progression classes			MMSE progression classes		
	N = 830	No progression N = 469	Slow progression N = 186	Fast progression N = 175	No progression N = 328	Slow progression N = 116	Fast progression N = 71	No progression N = 363	Slow progression N = 295	Fast progression N = 99
**CDR-SB Components at MCI diagnosis, mean (SD)**										
Memory	0.5 (0.2)	0.4 (0.2)	0.5 (0.2)	0.5 (0.3)	0.4 (0.2)	0.5 (0.2)	0.5 (0.2)	0.4 (0.2)	0.5 (0.2)	0.5 (0.2)
Orientation	0.1 (0.2)	0.1 (0.2)	0.1 (0.2)	0.2 (0.3)	0.1 (0.2)	0.1 (0.2)	0.2 (0.3)	0.1 (0.2)	0.1 (0.2)	0.2 (0.3)
Judgement/problem solving	0.2 (0.3)	0.1 (0.2)	0.2 (0.3)	0.3 (0.3)	0.1 (0.2)	0.2 (0.3)	0.3 (0.3)	0.2 (0.3)	0.2 (0.3)	0.2 (0.2)
Community affairs	0.1 (0.2)	0.0 (0.1)	0.1 (0.2)	0.2 (0.2)	0.0 (0.1)	0.1 (0.2)	0.2 (0.2)	0.1 (0.2)	0.1 (0.2)	0.1 (0.2)
Home and hobbies	0.1 (0.2)	0.0 (0.2)	0.1 (0.2)	0.2 (0.3)	0.0 (0.2)	0.1 (0.2)	0.2 (0.3)	0.1 (0.2)	0.1 (0.2)	0.1 (0.2)
Personal care	0.0 (0.1)	0.0 (0.0)	0.0 (0.1)	0.0 (0.2)	0.0 (0.1)	0.0 (0.0)	0.0 (0.2)	0.0 (0.1)	0.0 (0.1)	0.0 (0.1)

CDR-SB, Clinical Dementia Rating Scale-Sum of Boxes (scores for each component ranges from 0 to 3, with higher values indicating worse cognition); FAQ, Functional Activities Questionnaire; MCI, mild cognitive impairment; MMSE, Mini-Mental State Examination; SD, standard deviation.

Supplementary Table 5. MCI type at index among the overall sample and progression classes

	Overall sample	CDR -SB progression classes			FAQ progression classes			MMSE progression classes		
	N = 830	No progression N = 469	Slow progression N = 186	Fast progression N = 175	No progression N = 328	Slow progression N = 116	Fast progression N = 71	No progression N = 363	Slow progression N = 295	Fast progression N = 99
**MCI type, n (%)**										
Amnestic MCI - single domain	391 (47.1%)	225 (48.0%)	80 (43.0%)	86 (49.1%)	165 (50.3%)	60 (51.7%)	37 (52.1%)	175 (48.2%)	138 (46.8%)	46 (46.5%)
Amnestic MCI - multiple domain	319 (38.4%)	175 (37.3%)	77 (41.4%)	67 (38.3%)	116 (35.4%)	45 (38.8%)	26 (36.6%)	130 (35.8%)	123 (41.7%)	42 (42.4%)
Non-amnestic MCI - single domain	86 (10.4%)	52 (11.1%)	21 (11.3%)	13 (7.4%)	38 (11.6%)	4 (3.4%)	8 (11.3%)	44 (12.1%)	23 (7.8%)	5 (5.1%)
Non-amnestic MCI - multiple domain	34 (4.1%)	17 (3.6%)	8 (4.3%)	9 (5.1%)	9 (2.7%)	7 (6.0%)	0 (0.0%)	14 (3.9%)	11 (3.7%)	6 (6.1%)

CDR-SB, Clinical Dementia Rating Scale-Sum of Boxes; FAQ, Functional Activities Questionnaire; MCI, mild cognitive impairment; MMSE, Mini-Mental State Examination

Supplementary Table 6. Sample characteristics for MMSE progression classes

	MMSE progression classes		
	**No progression N = 363**	**Slow progression N = 295**	**Fast progression N = 99**
**Socio-demographic characteristics**			
Age (y), mean (SD)	76.3 (8.2)	80.0 (8.2)	79.3 (10.1)
Male, n (%)	162 (44.6)	118 (40.0)	44 (44.4)
At least some college education, n (%)	312 (86.0)	215 (72.9)	58 (58.6)
Non-Hispanic white, n (%)	309 (85.1)	217 (73.6)	63 (63.6)
Married, n (%)	216 (59.5)	147 (49.8)	60 (60.6)
Live with spouse/partner, n (%)	213 (58.7)	144 (48.8)	59 (59.6)
**Clinical characteristics**			
BMI, mean (SD)	26.4 (4.8)	25.6 (5.0)	26.4 (5.3)
First-degree family member with cognitive impairment, n (%)	237 (65.3)	173 (58.6)	61 (61.6)
At least one copy of the *APOE ε4* gene, n (%)	119 (32.8)	115 (39.0)	50 (50.6)
Any FDA-approved AD medication use, n (%) ‡	23 (6.3)	23 (7.8)	15 (15.2)
**Selected comorbidities**			
Hyperlipidemia, n (%)	227 (62.5)	187 (63.4)	64 (64.6)
Hypertension, n (%)	219 (60.3)	212 (71.9)	75 (75.8)
Depression, n (%)	142 (39.1)	96 (32.5)	32 (32.3)
Neuropsychiatric disorders, n (%)	66 (18.2)	43 (14.6)	11 (11.1)
Coronary artery disease, n (%)	45 (12.4)	47 (15.9)	13 (13.1)
Diabetes, n (%)	43 (11.8)	45 (15.3)	21 (21.2)
Atrial fibrillation, n (%)	38 (10.5)	31 (10.5)	8 (8.1)
Traumatic brain injury, n (%)	32 (8.8)	20 (6.8)	4 (4.0)
Sleep disorders, n (%)	34 (9.4)	11 (3.7)	3 (3.0)
Cancer, n (%) †	16 (4.4)	7 (2.4)	6 (6.1)
Congestive heart failure, n (%)	10 (2.8)	16 (5.4)	5 (5.1)
**Questionnaire/scale statistics**			
CDR-SB score at MCI diagnosis, mean (SD)	0.8 (0.7)	1.0 (0.8)	1.1 (0.9)
FAQ score at MCI diagnosis, mean (SD)	1.2 (2.4)	1.7 (3.0)	2.5 (3.6)
MMSE score at MCI diagnosis, mean (SD)	28.9 (1.2)	27.3 (1.6)	24.6 (3.0)
CDR-GS score at MCI diagnosis, mean (SD)	0.4 (0.2)	0.5 (0.1)	0.5 (0.1)
NPI-Q score at MCI diagnosis, mean (SD)	1.6 (2.4)	1.8 (2.9)	1.9 (3.2)
GDS score at MCI diagnosis, mean (SD)	1.8 (2.2)	2.2 (2.5)	2.4 (2.4)
Able to live independently, n (%)	344 (94.8)	265 (89.8)	83 (83.8)
**Follow-up**			
Years of follow-up, mean (SD)	4.0 (2.7)	3.5 (2.2)	2.9 (1.9)
Number of NACC visits pre- and post-index date, mean (SD)	7.5 (2.9)	7.0 (2.7)	6.8 (2.3)

‡FDA-approved AD medications include donepezil, galantamine, memantine, and rivastigmine.
†A high proportion of participants in the overall sample (77.1%) had unknown cancer status.

AD, Alzheimer's disease; *APOE* ε4, ε4 allele of the apolipoprotein E gene; BMI, body mass index; CDR-GS, Clinical Dementia Rating Scale-Global Score (scores range from 0-3, with higher values indicating worse cognition; all patients had a score ≤0.5 at the MCI diagnosis); CDR-SB, Clinical Dementia Rating Scale-Sum of Boxes; 0-18, higher values indicate worse cognition; all participants had a score ≤4 at the MCI diagnosis; CDR-GS, Clinical Dementia Rating Scale-Global Score; 0-3, higher values indicate worse cognition; all participants had a score ≤ 0.5 at the MCI diagnosis; FAQ, Functional Activities Questionnaire; 0-30, higher values indicate worse function; FDA, Food and Drug Administration; GDS, Geriatric Depression Scale (scores range from 0-15, with higher values indicating more advanced depression); MCI, mild cognitive impairment; MMSE, Mini-Mental State Examination; 0-30, lower values indicate worse cognition; NPI-Q, Neuropsychiatric Inventory-Questionnaire (scores range from 0-36, with higher values indicating worse neuropsychiatric symptoms); SD, standard deviation.

Supplementary Figure 1. Observed individual and predicted growth trajectories for the 3-class MMSE model

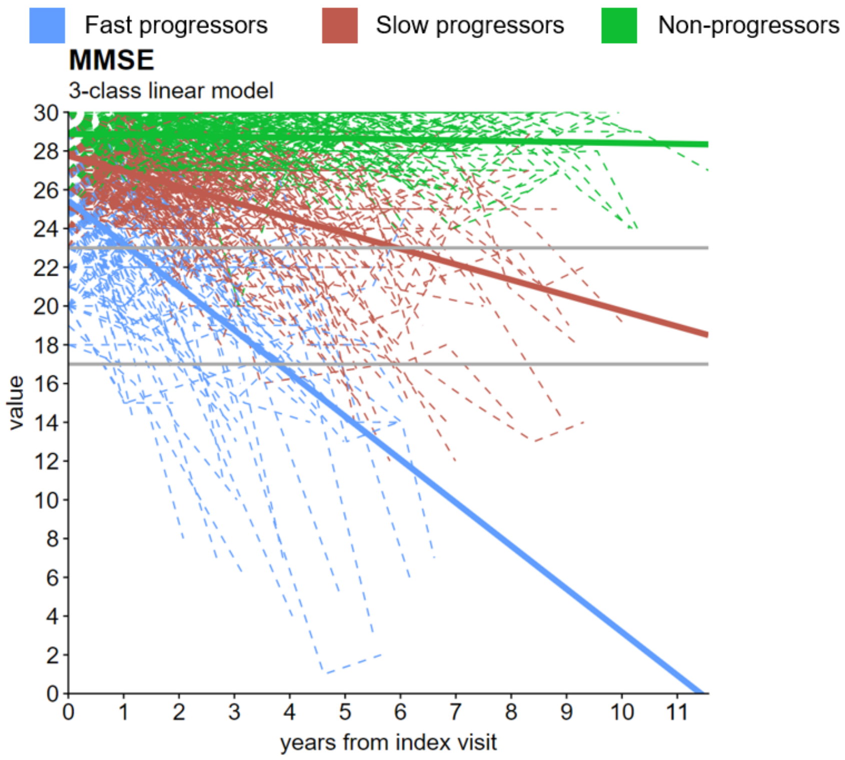

MMSE, Mini-Mental State Examination; MoCA, Montreal Cognitive Assessment.
[1] MoCA scores have been mapped into the MMSE scores using a published conversion algorithm (Trzepacz, 2015).
[2] Score ranges from 0 to 30. Lower values indicate worse cognition: 24 - 30 = No cognitive impairment; 18 - 23 = Mild cognitive impairment; 0 - 17 = Severe cognitive impairment.
[3] These models were run using the latent class mixed models (lcmm) package in R. Trajectories are based on lcmm's predictY function.

Supplementary Figure 2. Class assignment concordance between CDR-SB and MMSE, 3-class linear models

	N = 757
Same class assignment, N (%)	424 (56.0%)
Differing class assignment, N (%)	333 (44.0%)

**CDR-SB versus MMSE 3-class linear concordance**		**Number of participants per assigned classes based on CDR-SB**			
		**No progression**	**Slow progression**	**Fast progression**	**Total**
**Number of participants per assigned classes based on MMSE**	**No progression**	**278 (36.7%)**	61 (8.1%)	24 (3.2%)	**363**
	**Slow progression**	128 (16.9%)	**92 (12.2%)**	75 (9.9%)	**295**
	**Fast progression**	24 (3.2%)	21 (2.8%)	**54 (7.1%)**	**99**
	**Total**	**430**	**174**	**153**	**757**

21 **757**

CDR-SB, Clinical Dementia Rating Scale-Sum of Boxes; MMSE, Mini-Mental State Examination.
There were 757 participants who had both CDR-SB and MMSE scores available in the data.
